# Analyzability of Photoplethysmographic Smartwatch Data by the Preventicus Heartbeats Algorithm During Everyday Life: Feasibility Study

**DOI:** 10.2196/29479

**Published:** 2022-03-28

**Authors:** Steve Merschel, Lars Reinhardt

**Affiliations:** 1 Preventicus GmbH Jena Germany; 2 Institute for Applied Training Science Leipzig Germany

**Keywords:** photoplethysmography, wearable, smartwatch, heart rate monitoring, cardiac arrhythmia screening, atrial fibrillation, signal quality, activity profile

## Abstract

**Background:**

Continuous heart rate monitoring via mobile health technologies based on photoplethysmography (PPG) has great potential for the early detection of sustained cardiac arrhythmias such as atrial fibrillation. However, PPG measurements are impaired by motion artifacts.

**Objective:**

The aim of this investigation was to evaluate the analyzability of smartwatch-derived PPG data during everyday life and to determine the relationship between the analyzability of the data and the activity level of the participant.

**Methods:**

A total of 41 (19 female and 22 male) adults in good cardiovascular health (aged 19-79 years) continuously wore a smartwatch equipped with a PPG sensor and a 3D accelerometer (Cardio Watch 287, Corsano Health BV) for a period of 24 hours that represented their individual daily routine. For each participant, smartwatch data were analyzed on a 1-minute basis by an algorithm designed for heart rhythm analysis (Preventicus Heartbeats, Preventicus GmbH). As outcomes, the percentage of analyzable data (PAD) and the mean acceleration (ACC) were calculated. To map changes of the ACC and PAD over the course of one day, the 24-hour period was divided into 8 subintervals comprising 3 hours each.

**Results:**

Univariate analysis of variance showed a large effect (η_p_^2^> 0.6; *P*<.001) of time interval (phase) on the ACC and PAD. The PAD ranged between 34% and 100%, with an average of 71.5% for the whole day, which is equivalent to a period of 17.2 hours. Between midnight and 6 AM, the mean values were the highest for the PAD (>94%) and the lowest for the ACC (<6×10^-3^ m/s^2^). Regardless of the time of the day, the correlation between the PAD and ACC was strong (*r*=–0.64). A linear regression analysis for the averaged data resulted in an almost perfect coefficient of determination (*r*^2^=0.99).

**Conclusions:**

This study showed a large relationship between the activity level and the analyzability of smartwatch-derived PPG data. Given the high yield of analyzable data during the nighttime, continuous arrhythmia screening seems particularly effective during sleep phases.

## Introduction

### Background

Stroke is a leading cause of mortality and disability resulting in considerable economic costs of treatment and poststroke care [1]. With 5.5 million deaths in 2016, stroke was recognized as the second leading cause of death globally, after ischemic heart disease [1]. In the European Union, the number of people living with stroke is estimated to increase by 27% between 2017 and 2047, mostly due to population aging and improved survival rates [2]. Nevertheless, the constantly increasing burden of stroke also implies inadequate implementation or effectiveness of primary prevention strategies [1]. There is no doubt that besides targeting behavioral risk factors, effective screening for conditions that raise stroke risk, such as hypertension, diabetes mellitus, and atrial fibrillation (AF), is essential [1]. In that respect, particular attention must be paid to AF since it represents one of the main causes of stroke [3] and the most common sustained cardiac arrhythmia in adults, affecting between 2% and 4% of the general population [4]. Due to its paroxysmal and often asymptomatic (silent AF) clinical presentation, AF often remains undetected and is frequently only diagnosed upon a suffered stroke [5]. According to the guidelines of the European Society of Cardiology (ESC), 24- to 72-hour electrocardiography (ECG) is the standard of care to detect AF [4]. However, this is usually prescribed only when the patient is already experiencing symptoms (eg, palpitations, exertional shortness of breath, and chest pain), which is a strong indicator that the disease has already progressed to an increased severity (persistent, long-standing persistent, or permanent AF). In addition, the diagnostic yield of standard ECG monitoring is limited in the case of paroxysmal AF since prolonged (>7 days) observation periods are required [5]. Additionally, long-term monitoring using an insertable cardiac monitor is superior to traditional follow-up methods in detecting AF [6]. Thus, in patients over 65 years of age, routine self-monitoring of one’s pulse is a class I recommendation in the ESC guidelines for raising the suspicion of AF [4].

### Previous Work

Mobile health (mHealth) technologies enabling patient-initiated short-term or continuous long-term pulse recordings are thought to have extraordinary potential to improve the care and management of AF and may yield a practical option to determine AF burden [4,7,8]. In principle, the currently available mHealth devices for AF screening can be divided into the following types: smartphones, smart bands or smartwatches, earlobe sensors, and handheld ECG devices [7]. Aside from handheld ECG devices, all of these devices are based on photoplethysmography (PPG), which is an optical measurement technique for the detection of blood volume changes in the microvasculature [9]. PPG sensors are applied on the surface of the skin, where a light source illuminates the tissue that in turn scatters and partially absorbs the emitted light [10]. A photosensitive diode is used to measure variations in scattered light intensity attributed to cardiac synchronous changes in the blood volume with each heartbeat, resulting in the typical pulsatile component of the PPG waveform [9,10].

The evaluation of the diagnostic performance of various mHealth devices for AF detection revealed the highest sensitivity (95% to 98%) and specificity (95% to 99.6%) for smartphones and their associated applications [7]. Accordingly, both the Preventicus Heartbeats algorithm (PHA, Preventicus GmbH), which is a certified medical device (class IIa, CE marked), as well as the competing product FibriCheck (Qompium NV), showed excellent performance for smartphone recordings [11-14]. However, the diagnostic accuracy of smart bands and smartwatches was highly variable between studies and strongly dependent on the test conditions [7]. This is because PPG measurements are vulnerable to artifacts caused by contact pressure, skin tone, user movement, or bright ambient light [7,9,15]. In addition, the signal can also be affected by physiologic variations such as vasoconstriction, coughing, a deep gasp, or a yawn [10]. Hence, the best data quality is to be expected when subjects are sleeping or sitting still [7]. For 1-minute measurements using wrist-worn devices in a controlled laboratory setting (relaxed sitting position with both arms resting on a firm surface), both algorithms (PHA and FibriCheck) demonstrated sensitivity and specificity comparable to smartphone analyses [16,17].

### This Study

Against this background, the question arises whether PPG-based smartwatch measurements provide a sufficient data basis for noninvasive continuous AF screening. The PHA performs an automatic signal quality check before rhythm analysis and outputs the percentage of exploitable minutes [11]. Based on this, the present study aimed to evaluate the analyzability of smartwatch-derived PPG data generated by the PHA during everyday life and to determine the relationship between analyzability and activity level in adults who are in good cardiovascular health. Furthermore, this study aims to determine when it is worthwhile to screen for AF, which will help to save data and increase the life of the device battery.

## Methods

### Participants and Procedures

A total of 41 (19 female and 22 male) adults with an average age of 43.2 (SD 15.8; range 19-79) years volunteered to participate in this investigation. Excluded from the study were shift workers, those who were unable or unwilling to give informed consent, and patients already diagnosed with a cardiovascular disease, especially heart rhythm disorders (eg, AF or ectopic beats). Each participant was asked to continuously wear a smartwatch for a period of at least 24 hours while following their individual daily routine. Thus, the measurements comprised the whole span of everyday life situations ranging from nonphysical activities, like sleeping or office work, to physically demanding activities, such as sports.

### Ethics Approval

The study was conducted according to the Declaration of Helsinki. Informed consent was obtained after verbal and written explanation of the experimental design. This investigation provided the technical preparation for our Clinical Trial “Determine AF Burden with PPG Trial,” which was approved by the Northwest and Central Switzerland Ethics Committee (Project-ID: 2020-01983)

### Data Acquisition and Processing

**Figure 1:**
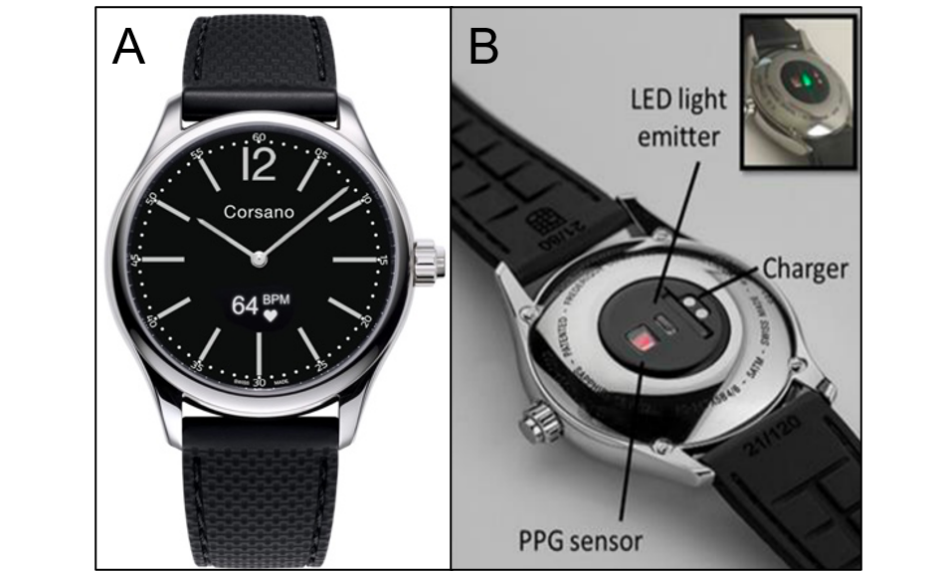
The smartwatch used for measuring photoplethysmographic (PPG) and accelerometric data. (A) A photograph of the front side of the wearable used in the study (Cardio Watch 287, Corsano Health BV). (B) The back side of the device indicating the exact position of the light-emitting diode (LED), the PPG sensor, and the charging contacts.

The PPG and mean acceleration (ACC) signal during everyday life were measured at the wrist using a smartwatch (Cardio Watch 287, Corsano Health BV). In total, 3 watches of the same type were used for data collection. Besides a 3D accelerometer (sensor range ±16 g), the device was equipped with a single channel PPG sensor module. A total of 2 green light-emitting diodes (LEDs; peak wavelength of 525 nm and maximum current of 30 mA) served as light sources for the sensor (Figure 1). The smartwatch measured PPG and accelerometric data with a sampling rate of 25 Hz.

Data transfer and storage was performed via a wireless connection (Bluetooth Low Energy, version 5.0) to a paired and preconfigured smartphone (Galaxy A40, Samsung Electronics Co Ltd) with the Android 10 operating system (Google LLC) that was running a data acquisition application provided by MMT. To avoid data loss, the internal memory (8 MB) of the Corsano smartwatch enabled data buffering for a period of about 24 hours. The PPG and ACC signal were automatically saved as JSON files on the smartphone and manually transferred to a desktop PC after the participant completed the measurement period. The raw data were further processed in MATLAB R2016a (MathWorks Inc). First, all data sets were manually inspected for completeness or potential data gaps. Since the smartwatch did not have an algorithm capable of automatically detecting when the watch was not worn, these phases were eliminated afterwards using custom-written programs in MATLAB. These periods were easy to identify as they appeared as Gaussian white noise in the raw data. Data sets containing more than 10% (2.4 hours) of erroneous data were excluded from the analysis. For each participant, a period of 24 hours was analyzed on a one-minute basis using the PHA. Consequently, a maximum of 1440 minutes (data points) was analyzed for each participant. To map changes over the course of a day, the 24-hour period of a day was divided into 8 equal subintervals (P1 to P8) of 3 hours or 180 minutes. The phases should divide the 24-hour day as sensibly as possible, thus resulting in the following phases: P1 (midnight to 3 AM; sleeping phase), P2 (3 AM to 6 AM; sleeping phase), P3 (6 AM to 9 AM; wake-up phase), P4 (9 AM to noon; working phase), P5 (noon to 3 PM; working phase), P6 (3 PM to 6 PM; working phase), P7 (6 PM to 9 PM; coming to rest phase), and P8 (9 PM to midnight; going to sleep phase). We have assumed that most people tend to behave roughly according to these time windows. Furthermore, on the one hand, one needs a division that is sufficiently large to be suitable for statistical tests. On the other hand, this division should not be too fine because otherwise, one obtains error probabilities that are too small with the Bonferroni correction for multiple testing. For this purpose, a compromise had to be found.

### Outcomes

**Figure 2:**
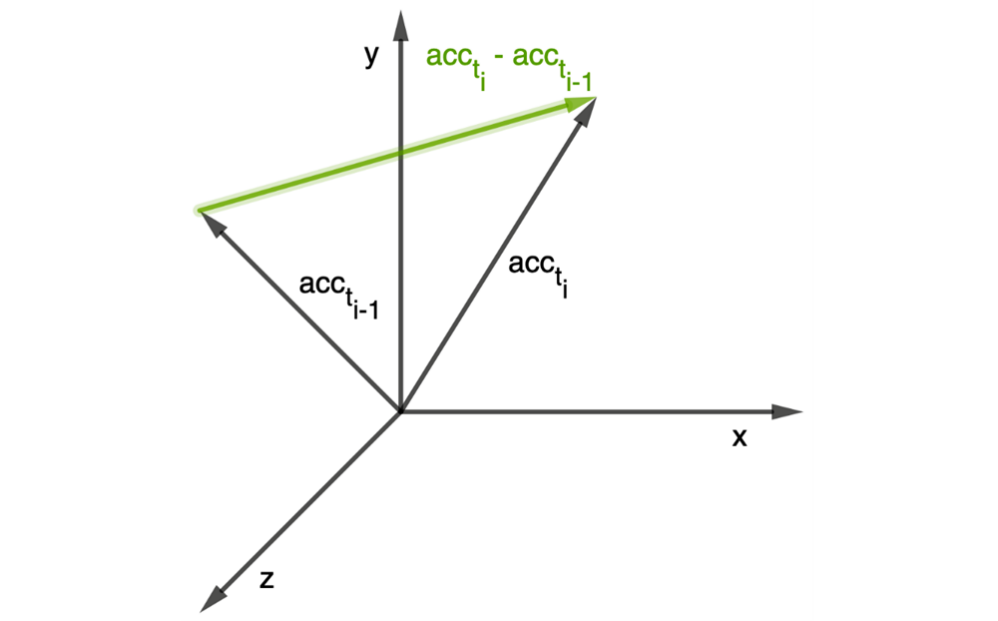
A visualization of the method for quantifying the activity level (represented by the ACC) of a participant. The length of the vector between 2 successive triaxial acceleration values (green arrow) was calculated using the Euclidean norm. ACC: mean acceleration.

The percentage of analyzable data (PAD) described the ratio between the number of measured PPG minutes and the number of exploitable minutes for the PHA. Exploitable minutes are determined by using the signal-to-noise ratio and the ACC data. For example, data sections disturbed by movement can be detected and marked. If the ratio of disturbed data sections to data sections with sufficient quality is more than 10%, then the entire minute is nonexploitable. A minute must therefore contain no more than 6 seconds of disturbed data for it to be exploitable [11]. The activity level of the participant (represented by the ACC) was estimated using the triaxial acceleration. For each time increment, the magnitude of acceleration change was calculated using the Euclidean norm (ie, the square root of the sum of squares) (Figure 2). Finally, the standard deviation of this was calculated for each period (P1 to P8) and expressed as the ACC .

### Statistical Analysis

Statistical analyses were performed using SPSS, version 23 (IBM Corp). Results are presented as means with SD and 95% CI. Sex comparisons were verified with Student *t* tests for unpaired samples. Where appropriate, effect sizes were calculated as standardized mean differences (*d*) with values >0.2, >0.5, and >0.8, indicating small, moderate, and large effects, respectively. The effects of measurement time on activity level or percent analyzability of PPG data were verified using univariate repeated measurement analysis of variance (ANOVA). Following the findings of the sex comparisons (comparing the outcomes between males and females), the ANOVA was conducted irrespective of sex using the whole sample (N=41). Bonferroni post hoc tests were used to verify localized differences. Practical relevance was estimated using partial eta squared (η_p_^2^) with values ≥0.01, ≥0.06, or ≥0.14 indicating small, moderate, or large effects, respectively [18]. Pearson product-moment correlations were calculated to examine linear associations between activity level and the analyzability of PPG data. The following criteria were adopted for interpreting the magnitude of correlations (*r*) between measures: <0.2, trivial; 0.2 to 0.3, small; 0.3 to 0.5, moderate; 0.5 to 0.7, large; 0.7 to 0.9, very large; and 0.9 to 1.0, almost perfect.

## Results

The comparisons of means did not reveal differences between sexes (*P*>.10) and for the PAD (*P*>.30) in any of the time intervals (P1 to P8). The only effect on a moderate level (*d*=0.52) was found for ACC in P5. No effects (for ACC *d*<0.19) were found for the PAD in P1, P2, P5, and P6, as well as for ACC in P4. All remaining sex comparisons resulted in small effect sizes (0.20<*d*<0.45).

**Figure 3:**
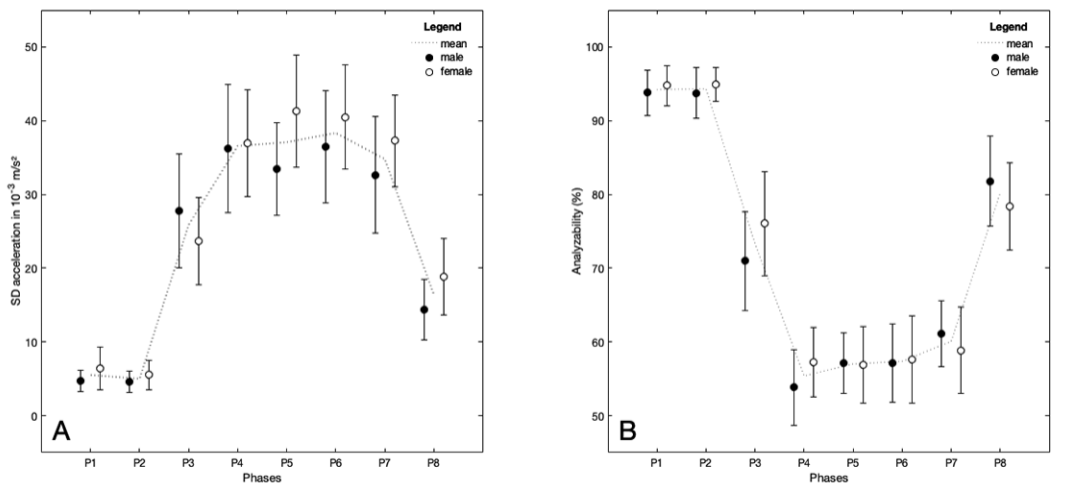
Activity level and analyzability of photoplethysmographic (PPG) data during a 24-hour period representing everyday life with respect to sex. (A) The average standard deviation of the acceleration (ACC). (B) The percentage of PPG data that could be analyzed for arrhythmia screening by the Preventicus Heartbeats algorithm. In both panels, mean values and 95% confidence intervals are symbolized by error bars. Data for males (n=22) and females (n=19) are depicted by filled or blank circles, respectively. The grey dotted lines represent the sex-independent mean.

In principle, the temporal changes in the PAD and ACC followed a characteristic but opposite trend throughout the day (Figure 3A and B) that roughly showed a u-shaped (PAD) or inverted u-shaped (ACC) pattern, respectively. The univariate ANOVA showed a large effect (η_p_^2^>0.6; *P*<.001) of the time interval on ACC and the PAD (Table 1). Accordingly, ACC values were significantly lower (*P*<.01) in P1 and P2 than in the remaining intervals (P3 to P8). This resulted in a PAD above 94% between midnight and 6 AM (P1 to P2; Table 1). In the time frame between 9 AM and 9 PM (P4 to P7), ACC values reached a plateau without significant changes (Table 1) on the highest level across the day (Figure 3A). During this period, the ACC average was approximately 37×10^−3^ m/s^2^. In accordance with ACC, there was little change in the PAD between 9 AM and 9 PM (P4 to P7). During this phase, values for this percentage ranged between 55% and 60%, on average. At the same time, these values represented the minimum over the span of a day. Moreover, the values of this parameter in P3 and P8 were clearly higher (73% to 80%), though not reaching the level of P1 and P2.

Interindividual variations in the PAD (SD<7%) and ACC (SD<5×10^-3^ m/s^2^) were lowest at night (P1 to P2) and highest around midday (P3 to P4; SD [ACC] 15×10^-3^ m/s^2^ to 18×10^-3^ m/s^2^; SD [PAD] 10% to 15%).

**Figure 4:**
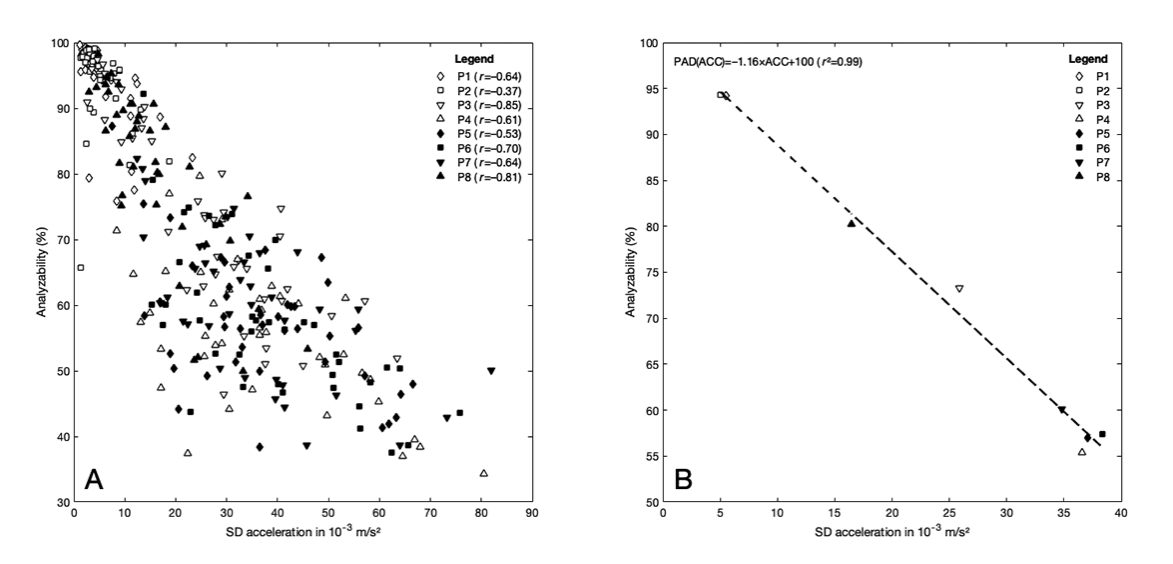
The relationship between activity level as expressed by the average standard deviation of the acceleration (ACC) and the analyzability of the photoplethysmographic data for arrhythmia screening generated by the Preventicus Heartbeats algorithm over 8 periods of a single day (P1-P8, compare to Figure 3). (A) Data from different time intervals are symbolized by diamonds, squares, and triangles, and Pearson product-moment correlations (*r*) are given for each period. (B) Mean values across all subjects for the ACC versus percentage of analyzable data (PAD) in each of the 8 periods. The dashed line represents the linear regression and the corresponding equation with the coefficient of determination (*r*^2^) is given.

The PAD and ACC showed an inverse correlation in all investigated periods (Figure 4A). Except for P2 (*r*=–0.37), all correlations were large (P1, P4, P5, P7) or very large (P3, P6, P8). The linear regression analysis of the averaged data resulted in an almost perfect coefficient of determination (*r^2^*=0.99; Figure 4B).

The values of the PAD ranged between 34% and 100%, with an average of 71.5% for the whole day, which is equivalent to a period of 17.2 hours.

**Table 1:** The average standard deviation of the acceleration and percentage of analyzable data during each phase of the day (N=41).

Outcome	Phase, mean (SD)									*P* value^a^		η_p_^2^		Bonferroni post hoc comparisons (*P*<.01)^b^
	P1^c^	P2^d^	P3^e^	P4^f^	P5^g^	P6^h^	P7^i^	P8^j^						
ACC^k^ in 10^-3^ m/s^2^	5.50 (4.70)	5.00 (3.70)	25.9 (15.3)	36.6 (17.4)	37.1 (15.3)	38.3 (16.0)	34.8 (15.8)	16.4 (10.1)	<.001		0.61		P1<P3; P1<P4; P1<P5; P1<P6; P1<P7; P1<P8; P2<P3; P2<P4; P2<P5; P2<P6; P2<P7; P2<P8; P3<P5; P3<P6; P4>P8; P5>P8; P6>P8; P7>P8	
PAD^l^	94.2 (6.30)	94.3 (6.50)	73.3 (14.9)	55.4 (10.7)	57.0 (9.82)	57.4 (12.0)	60.1 (11.0)	80.2 (13.2)	<.001		0.75		P1>P3; P1>P4; P1>P5; P1>P6; P1>P7; P1>P8; P2>P3; P2>P4; P2>P5; P2>P6; P2>P7; P2>P8 P3>P4; P3>P5; P3>P6; P3>P7; P4<P8; P5<P8; P6<P8; P7<P8	

^a^*P* value from the univariate analysis of variance.

^b^*P*<.01 for the comparison of each pair of phases.

^c^P1: midnight to 3 AM.

^d^P2: 3 AM to 6 AM.

^e^P3: 6 AM to 9 AM.

^f^P4: 9 AM to noon.

^g^P5: noon to 3 PM.

^h^P6: 3 PM to 6 PM.

^i^P7: 6 PM to 9 PM.

^j^P8: 9 PM to midnight.

^k^ACC: averaged standard deviation of the acceleration.

^l^PAD: percentage of analyzable data.

## Discussion

### Principal Findings

The present study demonstrates a strong relationship between activity level during a routine day and the analyzability of smartwatch-derived PPG data. In addition, a large effect of daytime on the ACC and PAD was found that was mainly based on differences between day and night.

The inverted u-shaped ACC pattern (Figure 3A) represents the rest-activity circadian rhythm of the persons investigated, which reflects the function of the circadian timing system [19]. Since people working in shifts were excluded from this study, it can be reasonably expected that all participants were sleeping and therefore hardly moving between midnight and 6 AM (P1, P2). In accordance with that, the average ACC values and the corresponding variations were minimal. By contrast, the ACC was the highest between 9 AM and 9 PM, which is equivalent to the main activity phase of the day for most people and typically comprises daily working as well as leisure activities. The comparatively high variability in the ACC data during this period can be explained by intraindividual differences in the load level and the temporal course of the everyday activities performed. Furthermore, in the 2 intervals (P3, P8), on an intermediate ACC level, differences in the times for waking up and going to bed are reflected. Since an almost perfect inverse linear relationship (*r*^2^=0.99) has been found between the ACC and the PAD (Figure 4B), all aforementioned statements for ACC also apply in an inverse manner for the PAD.

In line with other studies, it can therefore be concluded that movement strongly impacts the quality of the PPG signal [9,10,15]. During ambulatory monitoring, various types of motion (eg, walking, stretching, and finger tapping) are present that can cause periodic or nonperiodic motion artifacts (MA) [20]. Besides whole-body movements, the relative movement between the PPG sensor and human skin is a potential source of MA, as well. Therefore, it is essential to tighten the wristband of the smartwatch in such a way that the device cannot shift, but keep it loose enough to avoid cutting off blood circulation. Although all participants were instructed to do so in this investigation, it cannot be ruled out that in some cases, the device was not fitted correctly. This may be a potential explanation for the occurrence of some erroneous measurements during the night as well, besides nightly physical movement like going to the bathroom.

The reduction of MA remains a major challenge in processing PPG data since they can be in the same frequency range as the heart rate signal [21]. Although various signal processing methods have been proposed, satisfactory performance in removing or reducing MA has not yet been achieved [21]. Thus, efforts have been made to improve the performance of noise reduction algorithms through adaptations in sensor design. Nowadays, multichannel PPG measurement systems comprising multiple sensor modules with LEDs of different wavelengths or colors (ie, green, red, or infrared), constitute the standard across multiple models of current PPG-based smartwatches (eg, Polar Vantage series, Samsung Galaxy Watch3, Apple Watch Series 6, and Withings ScanWatch) [21]. Given this, the sensor module used in this study comprised of only 2 LEDs with one wavelength and one photodiode fails to comply with the current standard. Consequently, the PAD is likely significantly higher when measured with newer smartwatches. This hypothesis must be investigated in further studies.

However, the present findings identified a nocturnal time frame of 6 hours with an excellent PAD. Of the 360 minutes between midnight and 6 AM, on average, only about 20 minutes were not exploitable for the PHA, suggesting that AF screening may be particularly effective during this phase. This strategy is supported by several studies showing that AF typically occurs at night or in the early morning hours [22-25].

### Limitations

Some limitations must be mentioned. Firstly, the participants were mainly recruited from occupational groups performing office work. We assume that the participants have similar activity profiles (ie, sleep during P1 and P2, wake up during P3, active during P4 to P7, go to sleep during P8). The participants did not keep a diary detailing their activities. Furthermore, no actigraphy techniques were used, either. Other occupational groups such as craftspeople or postal workers might show clearly different activity patterns during the same time of day, which might have an impact on the analyzability of PPG data. In addition, our results only apply for weekdays and not for weekends, when activity profiles most likely look different, especially among the working population. Finally, this study only involved healthy participants and excluded patients with cardiovascular diseases. Hence, the present findings cannot be generalized to the overall older adult population. The analyzability of smartwatch-derived PPG data for a population with major disorders requires further investigation.

### Conclusions

This study showed a strong relationship between activity level and the analyzability of smartwatch-derived PPG data and a large effect of time of day on both parameters. That effect was mainly based on the differences between day and night. In conclusion, the present findings suggest that nocturnal AF screening may be particularly effective since the yield of analyzable data was the highest in the time interval between midnight and 6 AM. During this phase, around 94% of the PPG recordings had an appropriate signal quality for rhythm analysis, which is a crucial prerequisite for reliable screening for cardiac arrhythmias such as AF.

## Abbreviations

ACC: mean acceleration
AF: atrial fibrillation
ANOVA: analysis of variance
ECG: electrocardiography
ESC: European Society of Cardiology
LED: light-emitting diode
MA: motion artifacts
mHealth: mobile health
MMT: Manufacture Modules Technologies SA
PAD: percentage of analyzable data
PHA: Preventicus Heartbeats algorithm
PPG: photoplethysmography
